# Geomorphometric Methods for Burial Mound Recognition and Extraction from High-Resolution LiDAR DEMs

**DOI:** 10.3390/s20041192

**Published:** 2020-02-21

**Authors:** Mihai Niculiță

**Affiliations:** Department of Geography, Alexandru Ioan Cuza University, 700505 Iași, Romania; mihai.niculita@uaic.ro; Tel.: +40-742-824-349

**Keywords:** archaeological topography, tumulus, burial mound, geomorphometry, high-resolution, DEM, LiDAR, random forest

## Abstract

Archaeological topography identification from high-resolution DEMs (Digital Elevation Models) is a current method that is used with high success in archaeological prospecting of wide areas. I present a methodology through which burial mounds (tumuli) from LiDAR (Light Detection And Ranging) DEMS can be identified. This methodology uses geomorphometric and statistical methods to identify with high accuracy burial mound candidates. Peaks, defined as local elevation maxima are found as a first step. In the second step, local convexity watershed segments and their seeds are compared with positions of local peaks and the peaks that correspond or have in vicinity local convexity segments seeds are selected. The local convexity segments that correspond to these selected peaks are further fed to a Random Forest algorithm together with shape descriptors and descriptive statistics of geomorphometric variables in order to build a model for the classification. Multiple approaches to tune and select the proper training dataset, settings, and variables were tested. The validation of the model was performed on the full dataset where the training was performed and on an external dataset in order to test the usability of the method for other areas in a similar geomorphological and archaeological setting. The validation was performed against manually mapped, and field checked burial mounds from two neighbor study areas of 100 km^2^ each. The results show that by training the Random Forest on a dataset composed of between 75% and 100% of the segments corresponding to burial mounds and ten times more non-burial mounds segments selected using Latin hypercube sampling, 93% of the burial mound segments from the external dataset are identified. There are 42 false positive cases that need to be checked, and there are two burial mound segments missed. The method shows great promise to be used for burial mound detection on wider areas by delineating a certain number of tumuli mounds for model training.

## 1. Introduction

The LiDAR technology available in the 70s and 80s became a de facto standard for obtaining high-resolution DEMs of bare earth after the 90s [1,2,3,4]. Currently, these DEMs have at least regional or even national coverages in some parts of the world, and their usage through visualization and/or automatic/semi-automatic recognition of features has become a research direction that has broad implications in geosciences [5,6] or archaeology [7,8,9,10,11,12,13,14,15,16,17,18,19,20,21,22,23]. In archaeology, the usage of LiDAR is becoming a standard in archaeological mapping at regional [24] and national scale [25]. The availability of computation hardware and software, together with high-resolution DEMs, makes it possible and feasible to use this approach for different types of landforms, natural or anthropic.

The high-resolution DEMs obtained from LiDAR data can represent a source of information for the geomorphologist or the archaeologist, even under forests [10]. There are limitations [26], concerning technology but also the features that can be recognized. The visualization of the DEM, together with other geomorphometric variables, can increase the quantity of information that can be distinguished [27,28,29,30,31,32,33,34,35]. When the features that need to be identified are rather small, cover wide areas and are big in number, automatic or semi-automatic methods for their identification and delineation can improve the creation of inventories [36,37,38,39,40]. These considerations apply the best to archaeological features that appear as mounds or holes [41,42,43,44]. Some approaches rely solely on optical satellite imagery [32,45] or other types of geophysical data [46].

## 2. Study Area

**Figure 1:**
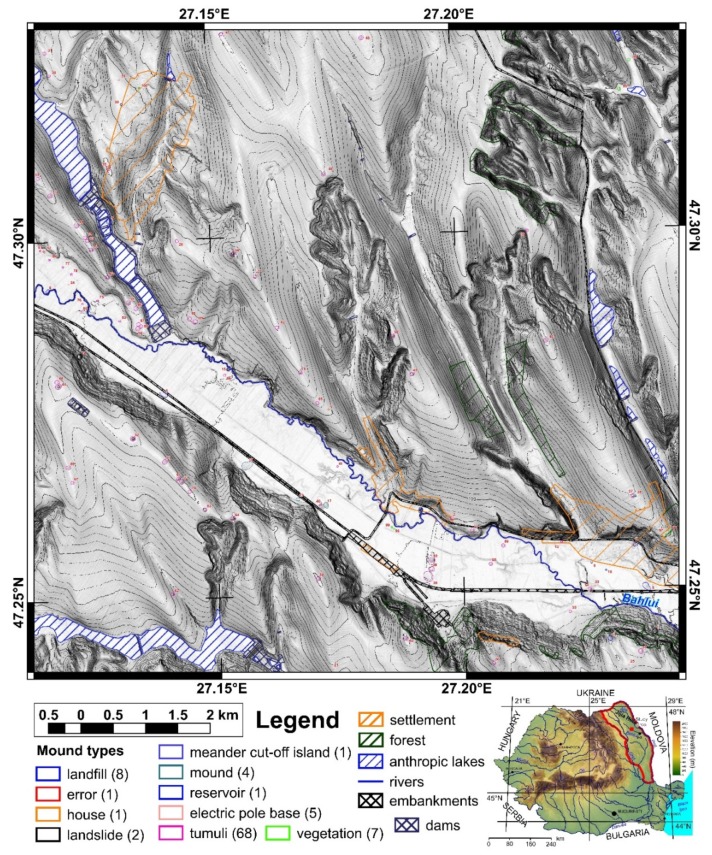
The geographic position of the northern study area and the location of the delineated mounds. (a high-resolution version can be found at https://doi.org/10.6084/m9.figshare.11798517.v2).

**Figure 2:**
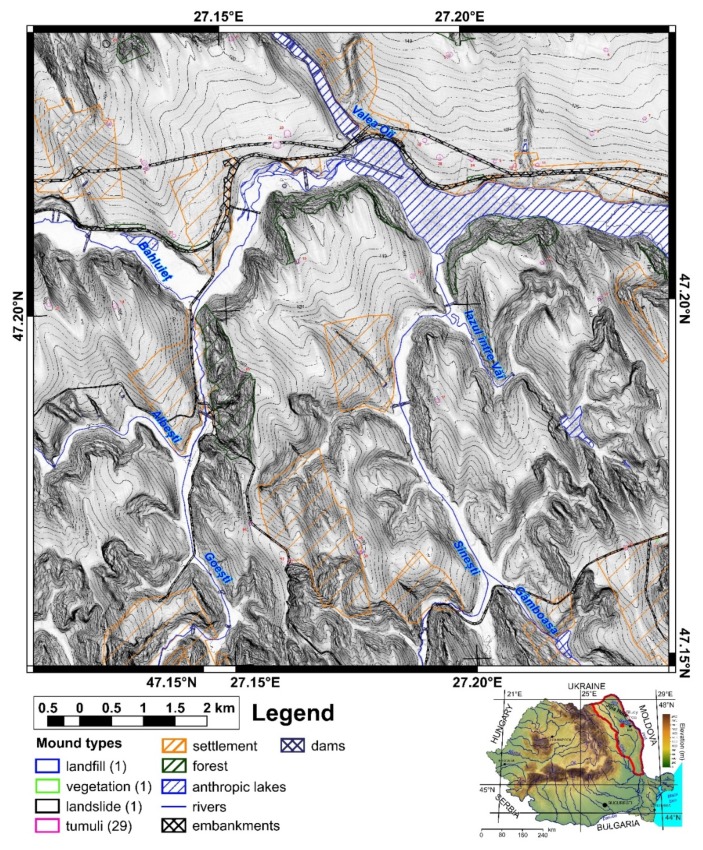
The geographic position of the southern study area and the location of the delineated mounds. (a high-resolution version can be found at https://doi.org/10.6084/m9.figshare.11798613.v2).

The study area is located in north-eastern Romania in the Jijia Hills (Figure 1 inset), a hilly area developed on monoclinic Miocene rocks. The hills are in general asymmetric, because of the monoclinal structure and of the homoclinic shifting of the channel network [47], which also developed wide floodplains (0.5–2 km width). Two neighbor square areas of the same size (100 km^2^) were chosen for training and validation (Figure 1 and Figure 2). The northern study area (Figure 1) was used for training and internal validation, while the southern study area (Figure 2) was used for external validation.

In the study area, the archaeological literature mentions a main period when burial mounds were built: Bronze Age (5450–2450 years BP), during which several cultures [48] constructed mounds for burial purposes: Jamnaya (5200–4450 years BP), Catacomb (4550–3950 years BP) and Srubnaya (3950–3250 years BP).

The authors in [49,50] are one of the first archaeologists to investigate burial mounds in the study area, at Glăvăneştii Vechi (Iaşi County) and Corlăţeni (Botoşani County) and to find graves and goods that attribute the burial mounds to Yamnaya culture. These Yamnaya burial mounds are present on a large geographical area, in Eastern and South-Eastern Europe [51,52] and appear in Moldavian Plateau on various landforms: ridges (Corlăţeni [50]; Movila Carului from Şuletea [53]), hillslopes (La Stadole [50]), lower fluvial terraces (Valea Lupului [50]) and on floodplains (Glăvăneştii Vechi [49]). The main characteristic of these burials is the presence of red ochre, which covered the dead body. Other Jamnaya and post-Jamnaya burial burials were described in the proximity of the study area by [53,54,55]. Very often, the Bronze Age burial mounds were used by Hallstatt, Sarmatic or Turanic populations for secondary burials [55,56,57,58].

In the Moldavian Plateau, burial mounds appear all over the area, with decreased densities at higher altitudes and in the Central Moldavian Plateau and Tutova Hills. The biggest density of burial mounds is in Jijia Hills. An extensive spatial inventory of mounds was made by [59] for Botoşani County. This inventory registers only the presence of a burial mound and sometimes the height of the mound, without mapping the spatial extension. A quick view on LiDAR data or satellite imagery for Botoşani County shows us that this inventory is not complete, many burial mounds not being identified in this inventory.

Burial mounds in the study area were first described in 1872 [60]. Their locations cover both forests and arable lands, ridges, hillslopes or floodplains, and their origin was attributed to humans. A particular impressive discovery is related to a mound eroded by Podriga river in 1811, which revealed a grave with important gold artifacts [60]. The discovery reveals the situation that local populations were aware of these discoveries and considered these mounds as treasure locations. It is not strange that many mounds bear the marks of old excavations in search of gold treasuries.

**Figure 3:**
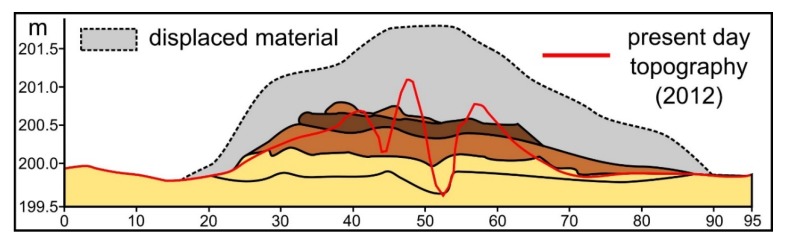
The stratigraphy of a burial mound (yellow to brown and grey) excavated by [61] at Corlăţeşti (Botoşani, County), redrawn after [61] with overlayed present-day LiDAR topography (red line); the location of this mound is represented in Figure 2 of [63], available in high-resolution at https://doi.org/10.1371/journal.pone.0227335.g002 or https://doi.org/10.6084/m9.figshare.11340419. (a high-resolution version can be found at https://doi.org/10.6084/m9.figshare.11798616.v1).

The investigation of [49,50,61] and other recent investigations [62] show that the present-day morphology of the burial mounds is not the same as when these features were constructed (Figure 3). Their initial shape was of a truncated cone. Natural and human-induced erosion tends to bury the basal edge of the structure and to flatten the upper part. Often, besides the burial of the basal edge, in this area, a concave surface appears because the terrain beside the mound is not plowed, and the last plow row is deposited in its vicinity. This situation makes their identification and delineation difficult in extreme cases, where the initial topography is heavily smoothed. In such cases, satellite images from the spring or autumn season, when the agricultural fields are tilled, could be used to identify the burial mounds [38], the soil developed on these features having lighter colors than the A horizon of the surroundings soils (chernozems and faeozems).

## 3. Materials and Methods

### 3.1. LiDAR Data

LiDAR data used in this study was acquired at the beginning of 2012 using a Leica ALS60 system by a company commissioned by Prut-Bârlad Water Administration. The original point cloud data (21,708 km^2^) in geographic coordinates on the ETRS89 datum (EPSG:4258) was classified by the producer using an unspecified algorithm and converted to ASPRS 1.1 format classes. Data georeferencing was realized using a network of 387 geodetic points, measured both in ERTS89 and Stereo 70 Marea Neagră data and for which a quasi-geoid model was computed. The LiDAR point density was between 2 and 6 points per 1 m^2^.

The point cloud was filtered to exclude vegetation and man-made features and to obtain a bare-earth DEM at 0.5 m spatial resolution. The result is a good bare earth DEM because the data was acquired in late winter, and the study area has low forest cover, but there are several areas where the algorithm for point cloud classification failed: in forests and on reservoir deltas were the lower vegetation is very dense, or in the built-up areas. The final DEM was resampled with a bicubic interpolator at 5 m spatial resolution.

### 3.2. Mound Manual Delineation and Field Recognition

First, using the LiDAR DEM, shading, slope, aspect, curvatures and contours in 2D and 3D views, different types of mounds were delineated as polygons that represent the basal edge of the mounds for the northern (98 mounds) and southern (32 mounds) study areas. The delineated mounds were latter checked on aerial imagery and in the field to assign an attribute (Figure 1 and Figure 2) as it follows for the northern study area: burial mounds (69), landfills (8), DEM interpolation error (1), non-filtered high vegetation features (7), wrongly classified LiDAR points (1), landslide body terrain rough features (2), meander cut-off island (1), mounds (3), water reservoir (1) and the electric pole base (5). For the southern study area, from 32 mounds, 29 are burial mounds while landfills, vegetation and landslide body are one mound per feature.

**Figure 4:**
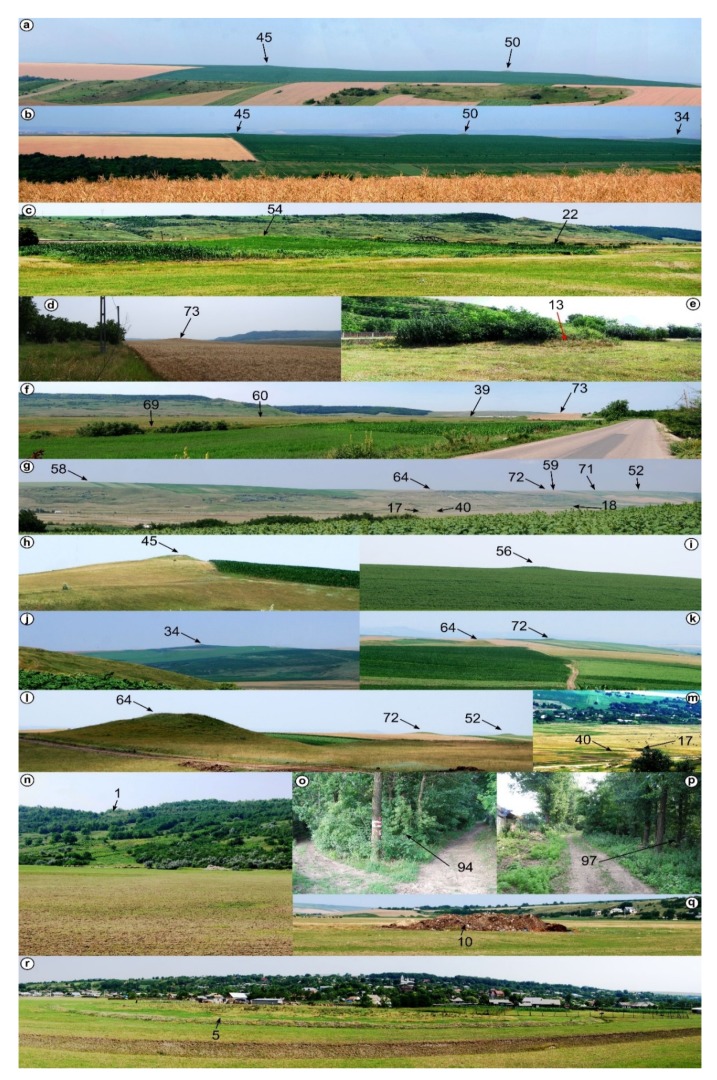
Field photos for verification (the numbers are the burial mound IDs from Figure 1): (**a**)—burial mounds 45 and 50 as seen from burial mound 36; (**b**)—burial mounds 34, 50 and 45 as seen from burial mound 46; (**c**)—burial mounds 22 and 54; (**d**)—burial mound 73; (**e**)—burial mound 13; (**f**)—burial mounds 39, 60, 69 and 73; (**g**)—burial mounds 40, 52, 58, 59, 64, 71 and 72 and mounds 17 and 18; (**h**)—burial mound 45 seen from the south; (**i**)—burial mounds 56; (**j**)—burial mound 34 seen from burial mound 62; (**k**)—burial mounds 64 and 72 seen from burial mound 58; (**l**)—burial mounds 64, 72 and 52; (**m**)—burial mound 40 and mound 17; (**n**)—landslide; (**o**,**p**)—vegetation; (**q**,**r**)—landfill. (a high-resolution version can be found at https://doi.org/10.6084/m9.figshare.11798625.v1).

Mounds are landforms created by different processes, but with similar morphology, a situation that can be associated with geomorphometric convergence, similar to the morphological convergence from biology [64]. The overall shape of the landform alone cannot always be used to specify exactly the process that created it. That is why all the burial mounds were verified in the field, and the classification from remote sensing images was validated (Figure 1 and Figure 2). Only two mistakes (delineated as burial mounds, found to be something else) were revealed by the field validation (IDs 35 and 94, in Figure 1, Figures S4 and S10). The validation was based not on geophysical or archaeological prospection but on the geomorphological observation of the morphology in the field. In general, the burial mound vs. non-burial mound distinction is easy to be done both on remote sensing images and in the field, especially for a trained person (both regarding the local geomorphology and archaeology of the study area). There are not many natural processes that can produce landforms with a shape similar to a burial mound. Humans can instead produce various types of mounds through the deposition of materials, and the type of material is an indication of the mound typology. I delineated anthropic burial mounds or natural features (vegetation not filtered from the point cloud and landslide body rough features only when these had similar shape and size with burial mounds). The best field pictures for showing the presence of a burial mound are taken from a neighbor ridge (Figure 4), especially in the case of smoothed burial mounds.

### 3.3. Geomorphometrical Methods

**Figure 5:**
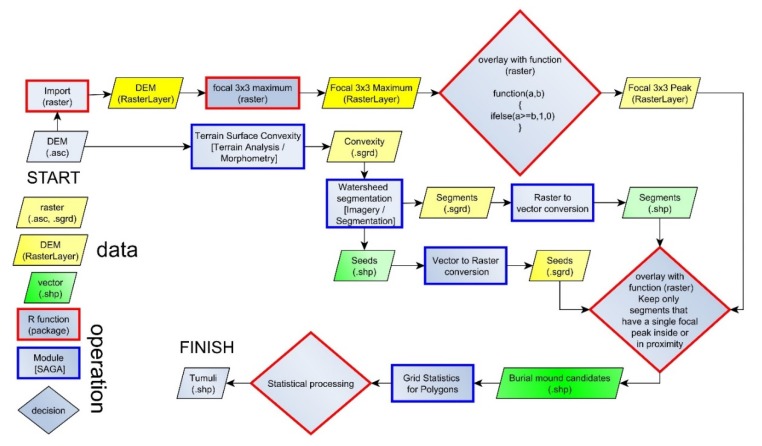
The proposed workflow. (a high-resolution version can be found at https://doi.org/10.6084/m9.figshare.11798634.v1).

Since geomorphometry deals with the quantitative depiction and analysis of Earth’s surface [65], the use of geomorphometrical variables [66] and methods of geomorphometrical object delineation [67] appears to be the right approach [68]. Supervised and unsupervised geomorphometrical methods for landform detection and delineation [67] are based either on statistical methods or on a priory model of process-form systems [69,70]. Their classification as pixels or objects is done depending on the composition of the final object that is delineated. I have used both types of geomorphometric object classification and delineation (Figure 5): a system of surface specific-points [71,72,73] and a statistical classification (Random Forest).

The peak extraction was performed using the search of the maximum value in a focal window. If the elevation value was the highest in the focal window, it was assigned as a peak. Twelve focal window sizes of square shape were tested: from 3 × 3 to 25 × 25. The method proposed by [71] can be easily implemented in any GIS software, which has scripting capabilities. We used the R statistics software [74], which is an open source software and has GIS and remote sensing capabilities through various packages [75,76,77,78].

The logic behind the method proposed by [71,73] was followed, but the implementation required a different step by step procedure since the framework for implementation is different. The maximum value of elevation in square kernel windows (3 × 3 to 25 × 25 pixels) was computed using the focal function from the raster package [77], and using a user-defined R function applied through the overlay function from the raster package we checked if the value of elevation for every pixel is higher or equal with the focal maximum. This is true for peaks and false for non-peaks. Peaks were flagged with value one and non-peaks with a 0 value.

Since the local peaks appear on many rugged features, local convexity is used to differentiate the burial mounds, which are essentially local convex features of a certain horizontal and vertical extension of elevation, from the dissimilar local peaks of the rugged landform surface. Local elevation maxima correspond with local convexity maxima, and the change in convexity is marking the mound edge. The mounds are different in scale compared to the small-scale ruggedness, the convexity varying on longer distances for mounds compared to other small-scale convex features. That is why in order to identify mounds, watershed segmentation [79,80,81,82] was applied for terrain local convexity computed by the SAGA GIS 7.4.0 [83] implementation of [84] algorithm in a 5 × 5 pixels window size, using a Laplacian filter with 8 pixels neighborhood (terrain analysis/morphometry/terrain surface convexity).

**Figure 6:**
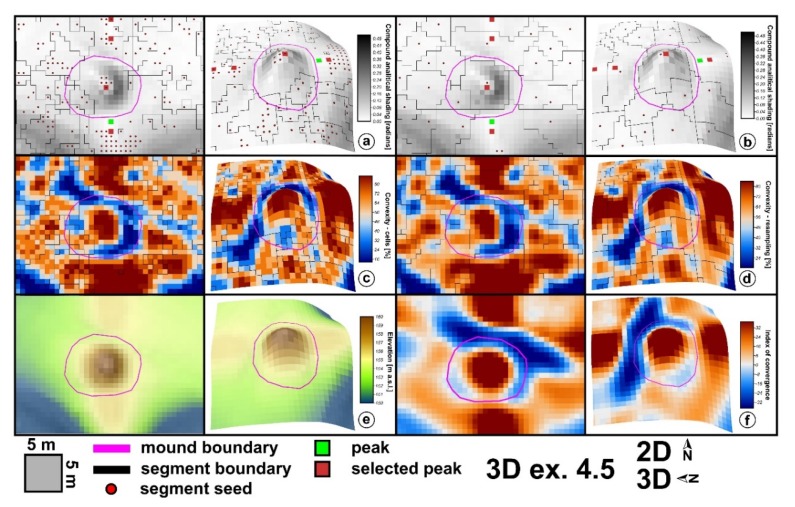
The geomorphometric data for burial mound 64 in 2D (left) and 3D (right): (**a**)—peaks, seeds and segments for local convexity computed with cell counting; (**b**)—peaks, seeds and segments for local convexity computed with cell count resampling; (**c**)—local convexity computed with cell counting; (**d**)—local convexity computed with cell count resampling; (**e**)—DEM; (**f**)—index of convergence. (a high-resolution version can be found at https://doi.org/10.6084/m9.figshare.11798637).

Local terrain convexity at every grid cell is expressed in percentage values of convex cells in a certain radius of cells [85]. I used a 5 × 5 area in order to smooth the local outliers, and in Figure 6, it can be seen that local peaks get 100% local convexity while the basal part of the mounds gets values close to 10%. True flat terrain will have 0% local convexity. The flat area threshold controls the 0% percent values. If 0 is chosen as the threshold, only no slope pixels will get this value. As the threshold increase, more areas will have 0% local convexity. Besides the implementation that computes the number of cells, which will give multiple maximum (100%) pixels on the maximum concavity (by counting cells), there is also a method that applies resampling to cell counting, and which will give only a maximum value pixel (Figure 6).

The watershed algorithm implemented in SAGA GIS [83] works in two phases. In the first phase, seeds are generated (which are either local minima or maxima), and the seeds are growing pixel by pixel until a certain threshold is reached or to simply put it if the digital surface changes its trend (from ascending to descending or vice-versa). Since the burial mounds are convex features, the local maxima were used for seed extraction.

The peaks obtained in the first step were refined through an R focal raster function that selected only the peaks that have in the 3 × 3 kernel window a local convexity seed, and are considered “selected peaks”. The local convexity watershed segmentation polygons resulted were kept, and considered burial mound candidates based on the condition that contain at least a single selected peak.

Besides the mentioned geomorphometrical variables, I also computed a wide range of variables described in Table S1. These variables were chosen considering that their values can describe the geomorphometric signature of burial mounds, by visual inspection and by boxplot comparison. The slope was computed by the [86] implementation of [87] slope algorithm in a 5 × 5 pixel window size and constraint on the central cell in SAGA GIS [83]. I have chosen this algorithm because it is smoothing the slope values, emphasizing the peaks. The full set of curvatures was computed from the same implementation. Negative openness and positive openness (morphometric protection index) were computed using the multiscale implementation of [88] algorithm from SAGA GIS in degrees difference from nadir as units. The radial limit of 100, with a multiscale factor of 3 and 8 sectors were used in order to emphasize local peaks. The index of convergence [89,90] was computed as aspect difference to the center cell in a 10 × 10 pixel window, emphasizing local peaks with local prominence. The real surface area was also considered, beside terrain ruggedness index [91], vector ruggedness measure and terrain texture [84] as measures of roughness. Wind exposition index [92,93] as dimensionless index show shadowed vs. exposed areas to the wind, while topographic position index [94,95,96], as a multi-scale implementation in SAGA GIS, pinpoints with high positive values local peaks. Upslope and downslope curvatures [97] computed using the gradients of the neighbor pixels in upslope and downslope contributing area computed by multiple flow direction, define local convexities. Flow accumulation, flow path length, slope length, valley depth [98] (as the height above downslope area) and cell balance [99] with low values and a minimum centered on the peak also pinpoint local convexities. Topographic wetness index [98,100] in its SAGA GIS implementation has local minima on mounds.

Shape descriptors computed by SAGA GIS [101,102] were also included as morphometric variable of the segments: area (A), perimeter (P), interior edge ratio (P/A), equivalent projected circle diameter (2 × sqrt(A/π)), sphericity (P/(2 × sqrt(A × π))), shape index (the inverse of sphericity), maximum diameter (Dmax) and Feret diameters. The ratio between the diameter of the local convexity watershed segment and its difference in elevation was computed as a measure of mound geometry. Besides the SAGA shape descriptors, compactness, form factor, roundness and elongation were computed.

### 3.4. Statistical Processing and Testing

The burial mound candidate watershed segments kept using the condition to contain a selected peak were further classified using a statistical method. The descriptive statistics (minimum, maximum, sum, mean, median, standard deviation, range, variance and multiple of 5 percentiles) of the geomorphometrical variables mentioned in Section 3.3 were derived for every watershed candidate segment by the SAGA GIS module Grid Statistics for Polygons. 

The Random Forest (RF) algorithm [103] implemented in R statistical software [74] as the randomForest package [104] was used to classify the burial mounds from the burial mounds local maxima watershed segment candidates. The random decision forests method for classification is a versatile machine learning algorithm, that has some desirable properties: does not overfit, is accurate by being less prone to noise-induced errors, and can be used with high dimensional and massive data with weak inputs [103,105]. The classification problem, in our case, is a binary one and imbalanced.

The RF model for classification performs five steps [103,105,106]: (i) first n_tree_ bootstrap samples are randomly sampled with replacement from the training dataset (with 1/3), (ii) second, for each bootstrap sample an unpruned classification tree is grown, at each node of the tree, randomly sampled m_tree_ of the predictor variables being used to choose the best split, (iii), third, prediction is based on the aggregation of the predictions of the n_trees_ by majority of votes or specified cutoff, (iv), forth, estimate error based on aggregation of the out-of-bag error (OBB—error computed from predicted data on the data not in the bootstrap sample) and (v) fifth, rate the variable importance based on how much prediction error increases when that variable is not considered.

The RF model parameters were derived with tuning functions from randomForest [106] and randomForestSRC [107,108,109] packages, while randomForestExplainer [110] and pdp [111] packages were used to visualize the results of the model. The mtry and node size tuning was performed using the tune function from randomForestSRC package. For selecting the best performing variables, cross-validation was used with the functions rfcv from randomForest [104] and boruta from Boruta [112] R statistical software packages. Based either on the importance or on the Boruta methodology, the most important variables are selected.

The cutoff parameter was used to set the probability used for the classification. Since the positive class (burial mounds) has a low proportion in the dataset (0.6%), I computed the cutoff probabilities as frequencies of the classes. Besides this, the classwt parameter was used, which favors one class or the other. The imbalanced two-class problem solutions implemented by [107,108,109,113] in randomForestSRC package were also used.

The setup of the training and validation schema was designed in order to prepare for a future application to the entire Moldavian Plateau region. The training was performed on the tumuli candidate segments dataset of the northern study area and validated internally on the whole dataset of tumuli candidate segments of the northern study area. The external validation was done on the dataset of segments from the southern study area. This setting of training assumes that by mapping a set of burial mounds from the entire area, fitting the model on them will give a certain prediction power when applied for the entire area. The training was done on a subset by conditioned Latin hypercube sampling implemented in R stat chls package [114,115,116].

### 3.5. GIS Implementation and Reproducibility

The proposed workflow can be easily implemented in any GIS software, which has scripting capabilities. I have used the R statistics software [74], which is an open source software and has GIS and remote sensing capabilities through various packages [75,76,77,78]. One such package offers the possibility to run also SAGA functions [117]. I have created an R script, which can perform the extraction based only on the DEM. The script is available on Zenodo (https://doi.org/10.5281/zenodo.3628805), together with the burial mound delineation and segments (https://doi.org/10.5281/zenodo.3628795), while the DEM data is available by request.

## 4. Results

### 4.1. Burial Mound Geomorphometry

**Figure 7:**
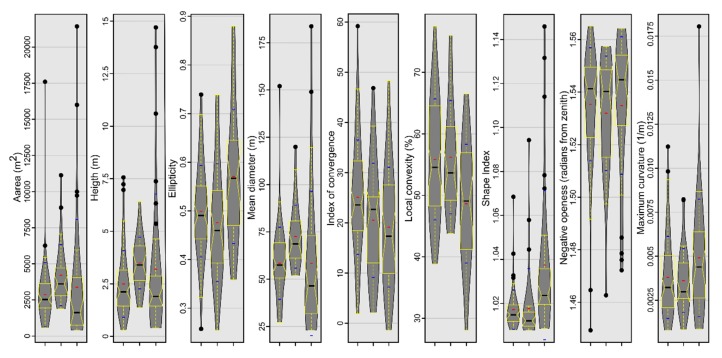
The descriptive statistics of burial mounds geomorphometry (black dots—outliers, black horizontal line—median, red horizontal line—mean, blue horizontal line—± standard deviation, grey polygon—kernel density, yellow boxplot with notch—interquartile range representing 50% of the data, upper part representing the 75th percentile, while the lower part the 25th percentile, lower whisker—minimum, upper whisker—maximum, notch—95% confidence interval of the median), for the fitting area (left, *n* = 68), for the testing area (center, *n* = 29) and other mound types geomorphometry (right, *n* = 33, for both the northern and the southern study areas). (a high-resolution version can be found at https://doi.org/10.6084/m9.figshare.11798646.v1).

The geomorphometric signature of the burial mounds can be assessed both by inspecting in 2D or 3D view the delineation overlayed with the geomorphometric variables and by boxplots that show the statistical distribution. In Figure 7, some geomorphometric variables of the delineated mounds are shown. Burial mounds from the northern study area (left in every boxplot from Figure 7) and those from the southern study area (center in every boxplot from Figure 7) have similar descriptive statistic measures, compared with the non-burial mounds. The most striking feature was the big number of outliers, but also the mean and distribution were significantly different from non-burial mounds versus burial mounds.

This situation can be explained by the fact that the non-burial mounds have shapes and morphology different from the burial mounds. While burial mounds have a conical shape, non-burial mounds might have several peaks and a not so smooth surface. Since they are newer, they also do not present the basal concavity (Figure 6 and the topographic cross-sections from Figures S1–S14) of the burial mounds, which is created mainly by tillage.

The most striking geomorphometric feature that was observed in the delineation work was the conic shape of the burial mounds, in this regard, the geomorphometric variables related to the convex curvature (local convexity, maximum curvature and index of convergence) and those related to local prominence being the ones that individualize the best these features (negative openness and hydrologic variables).

### 4.2. Burial Mound Peak Detection

The detection of the burial mounds starts from the idea that such features are typical geomorphometric peaks, local maxima of elevation. These peaks are local [118] but are evident at landform spatial resolutions of several decameters, and can be distinguished from the local noise. At the same time, local maxima, which have the geomorphometric conditions of being peaks at similar landform resolution with the burial mounds, are represented either by other man-made features (landfills and road embankments), by errors in the acquisition, classification or interpolation of LiDAR data or by other natural features. Such natural features are represented by the fragmented natural levees, river meander cut-off islands, ridges or landslide body rough topography. It is to be expected that the method will find many false positive features, which are not burial mounds. This is not necessarily a bad point of the methodology since the targeted morphology is identified. This would be reasonable if this number is low enough to allow reasonable manual inspection of the results.

**Figure 8:**
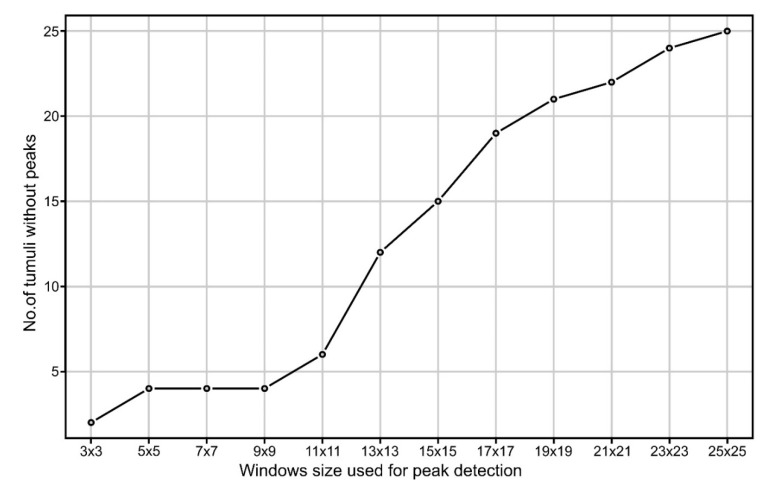
The variation of the number of delineated burial mounds without an identified peak as a function of the size of the window (in pixels) used for peak identification. (a high-resolution version can be found at https://doi.org/10.6084/m9.figshare.11798652.v1).

The use of the focal filters to identify maxima eliminates the local peaks located along hillslopes and emphasizes any local peak located on floodplains, plateaus or ridges. For the focal window sizes from 3 to 25 pixels, the following number of peaks were found for the northern study area: 31,543, 10,857, 5447, 3176, 2047, 1434, 1016, 795, 625, 504, 418 and 360. At a 3 × 3 focal window, for the northern study area from 69 burial mounds, only two (2.9%) did not have the corresponding identified peaks (burial mound 85 and 90 from Figure 1, Figures S9 and S10). This number increased to four (5.8%) at 5 × 5 up to 9 × 9 pixels focal window size, after which increased continuously toward 25 (32.2%) at a 25 × 25 pixels focal window size. Bigger focal window sizes decreased the number of peaks found inside the delineated burial mounds. The number of peaks was relatively stable between a 3 × 3 and 9 × 9 pixels windows size (Figure 8), after which their number decreased quickly. This means that the burial mounds had diameters between 15 and 45 m, which was consistent with the delineated burial mounds geomorphometry (Figure 7).

For the southern study area, 29,726 peaks were identified, one burial mound not having an associated peak. The burial mound with ID 25 was located on a hillslope and was cut by the national road embankment, due to smoothing having a shoulder rather than a peak.

Local convexity will smooth as the window size for computation increases, the 5 × 5 size being a trade-off that worked the best: only three burial mounds did not have peaks with seeds at 5 × 5 window size computation, while for wider window sizes the number would increase. Using a 3 × 3 size, the number of burial mounds that did not have peaks was also high, mainly because at that size, very local convexity was found, but the burial mounds were not delineated well as watershed segments.

Another issue appeared due to the local convexity computation method. When the cell count was used, there were many seeds (pixels with 100% local convexity), and only three burial mounds did not have selected peaks. However, the corresponding watershed segments did not delineate very well the burial mound, a situation that would influence the final step of statistical processing of the segments in order to derive the identified burial mounds. At the same time, using the seeds obtained from local convexity computed using the cell count resampling method for peak selection, 14 burial mounds would not have selected peaks identified. In order to resolve the issue, I used for peak selection the seeds obtained from local convexity computed with the cell count method and the watershed segments obtained from convexity computed with the cell count resampling method. These watershed segments delineate better the burial mounds.

For the northern study area from 31,543 peaks identified at a 3 × 3 pixels windows size, through the proposed automatic methodology, 26,220 were selected peaks. Two burial mounds were missed by the peak selection algorithm: burial mounds 55 and 76, and adding the other two missed because of peak inexistence (with ID 90 and 85), rose to four (6%) the number of missed burial mounds.

Regarding the other mounds identified by the manual mapping and field inspection, landslide mounds with IDS 1 and 14, and vegetation mound with ID 94 were not identified by the proposed method, while the others were identified.

For the southern study area from 29,276 peaks, 24,733 were selected, and 28 burial mounds had a selected peak. Burial mound with ID 24, which did not have a peak, was located on a hillslope and was cut by a road embankment, having a shoulder shape. Burial mound with ID 23 had selected peaks, but not on the watershed segment that was central to the mound.

### 4.3. Burial Mounds Segments Candidates

For the northern study area from 102,936 terrain convexity segments, 16,939 had corresponding peaks, from which 64 corresponded to real burial mounds, while 15,602 terrain convexity segments had at least a corresponding selected peak. The majority of the burial mound candidate segments were located along the Bahlui floodplain. Over the rest of the study area, these candidate segments were located mainly along the ridges and floodplains. For the southern study area from 103,741 terrain convexity segments, 15,840 had corresponding peaks and 14438 had corresponding at least one selected peak, from which 28 corresponded to real burial mounds.

Before running the RF classification, 2343 segments corresponding to anthropic lakes not masked from the LIDAR data were removed, together with very small (under 100 m^2^) and large (over 5000 m^2^) segments considered outliers (2456 segments), dams and anthropic levees (622 segments) and road or rail embankments (2753 segments). The filtering of the segments can be done further if there is knowledge about areas improbable to contain burial mounds (landslides, urbanized or forested areas). Finally, 12,745 burial mound segment candidates were selected for the classification step. For the southern study area, 10,606 burial mound segment candidates were selected for the classification step after eliminating segments corresponding to lakes (3726 segments), dams and anthropic levees (622 segments) and road or rail embankments (2753 embankments).

**Figure 9:**
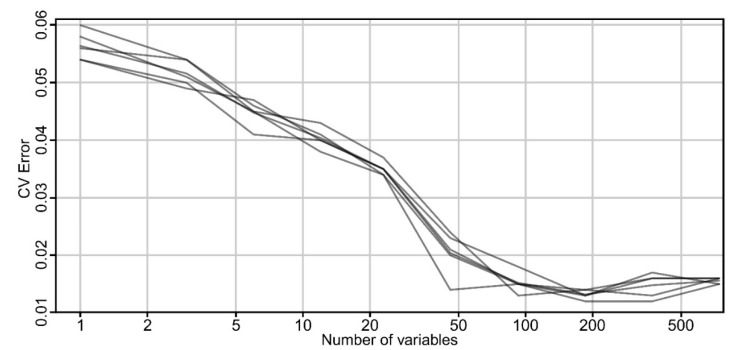
Plot of the CV error of 5 replicates for 10-folds rfcv with all the variables. (a high-resolution version can be found at https://doi.org/10.6084/m9.figshare.11798658).

First, the RF model was fitted with all the 751 variables (shape indices and descriptive statistics of elevation, local convexity, slope, maximum curvature and negative openness) computed as mentioned above. Ten-fold cross-validation (CV) results shows that using 183 variables, the error was the lowest (Figure 9). While RF can be used also for variable selection, based on its derived metrics, in our case lowering the number of input variables to the 10 best variables (chosen based on mean decrease in accuracy measure) actually gave worse results when the confusion matrix was investigated. This happens because in the RF model, if the node size is 1 (the n_trees_ are grown to their maximum depth), the model will create deeper trees, so overfitting will not happen, even though there are many variables. In this study case with extremely unbalanced class frequencies, the combination of a big number of trees and deeper trees will learn even from slight variations of variables that might appear to be correlated otherwise.

**Figure 10:**
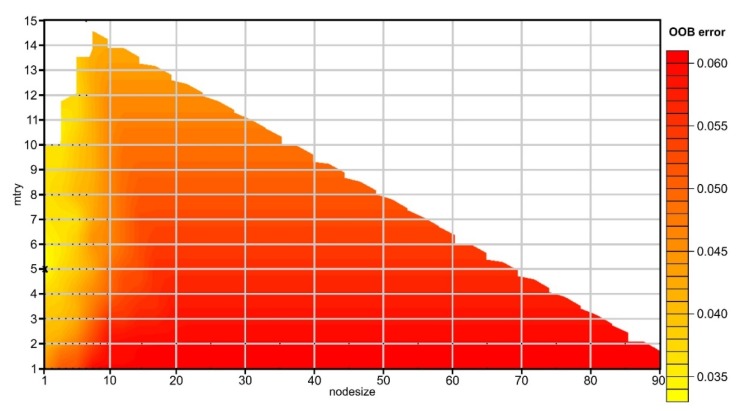
The plot of OOB error showing the mtry and node size tuning with 100 ntree. (a high-resolution version can be found at https://doi.org/10.6084/m9.figshare.11798661).

Three number of trees was tested (100, 1000 and 10,000), since a very big number does not necessarily improve the results, more important being the depth of the trees, but in the same time, the number needs to be high enough to get stable estimates of variable importance and proximity [106]. The results of the RF tuning show that mtry 5 and node size 1 gave the lowest OBB error (Figure 10) for up to 10,000 ntrees. In order to keep the computational part at a minimum and without losing accuracy, 100 ntrees were used. The main issue in this regard is that indeed OBB is the lowest, but in terms of accuracy matrix for our imbalanced problem, actually the mtry of 4 is better (Table 1).

In the present case, since the class frequencies were extremely imbalanced (burial mound class 0.6% for the northern study area and 0.2% for the southern study area), I chose to control this through the classwt parameter of the randomForest function from the randomForest package. This parameter will modify the majority vote at the node split, by replacing it with a vote fraction. I have used 0.01 for the non-burial mound class and 0.9, for burial mound class. This setup is giving better results both in terms of OBB error and confusion matrix than the 0.5 cut-off parameter of the randomForest function or the imbalanced RF implementation of [113] from randomForestSRC package.

The randomForest model had good accuracy, given by the OBB error at various training set sampling settings (Table 1), although the most accurate RF model in terms of OBB error was not necessarily the better in terms of the confusion matrix. The confusion matrix results I considered to give the final answer regarding the settings of the training dataset. Ideally, in the burial mound classification problem, the model should predict as many burial mounds possible while having a minimum of false positives.

**Figure 11:**
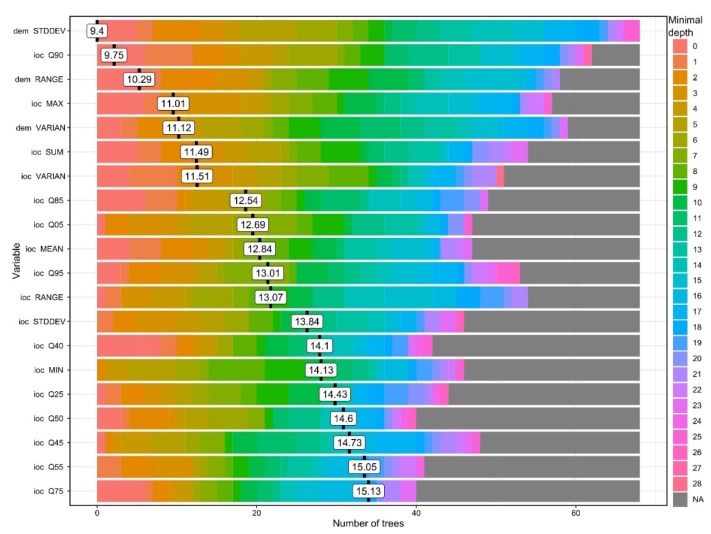
The plot of the minimal tree depth and its mean. (a high-resolution version can be found at https://doi.org/10.6084/m9.figshare.11798667.v1).

**Figure 12:**
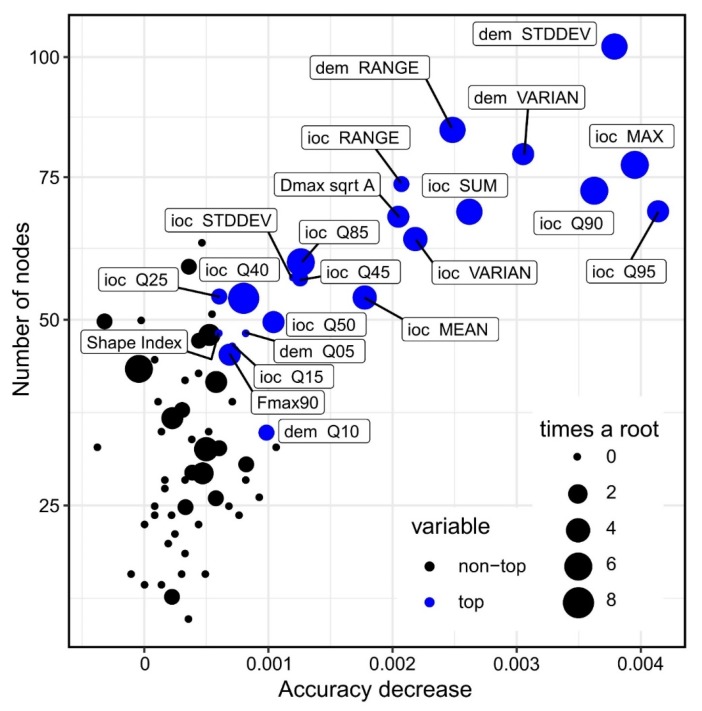
The plot of accuracy decrease versus the number of nodes and the times a root for all the variables. (a high-resolution version can be found at https://doi.org/10.6084/m9.figshare.11798673.v1).

Even at 1000 segments training dataset, 100 ntrees are enough for good results. I believe that this is happening at such a low number of trees because of the class imbalance, which is resolved with a small number of very deep trees. Regarding the variables that give the best confusion matrix results I applied an iterative approach in which each variable was tested for the number of true positives and false positives, and through this procedure the shape descriptors and the descriptive statistics of the altitude (referenced as dem in Figure 11 and Figure 12) and index of convergence (ioc) have given the best results, being used in fitting the final model. The list of the 72 used geomorphometric variables and shape descriptors is given in Table S2.

Investigating the minimal depth of the trees for each variable and its mean (Figure 11), it can be seen which are the variables that are the most predictive (the smallest mean value of minimal depth) in terms of tree topologies. From the 20 variables plotted in Figure 11 we can see that elevation standard deviation, 90th quartile of ioc and elevation range have the longest trees and are always used to split trees at the root. The RF model constructed 100 trees and no limit to the maximum number of terminal nodes trees in a tree was set up, in the Figure 11 legend it is visible that trees were split until a depth of 28.

Variable importance is given by the difference in the OBB error between the situation when only the variable is perturbed and when it is not, for that tree. In Figure 12 we can see plotted all the variables used, as a relation between accuracy decrease (if that variable is perturbed) and the number of node splits for that variable, the size of the circle indicating the number of times that the variable is a root. The blue-colored circles correspond to the first ten most important variables. We can see again here variables, which are also predictive and have a presence as the number of nodes and times a root. There is a clear relationship between the accuracy decrease and number of nodes (as the number of nodes decreases the accuracy is not so decreased), but there are also not so important variables (by accuracy decrease, mainly index of convergence percentiles), with a small number of nodes but used many times as a root. This situation shows that using these variables in the model and not removing them, although will increase the OBB error, will allow the model to increase the true positives and decrease the false positives (Table 1).

The variable interactions (in pairs of one by one) are given by the splits that appear in maximal subtrees in regard with the variables and can be assessed by computing the mean conditional minimal depth (a variable is taken as root/node, and the mean minimal depth is computed for the other variable) versus mean unconditional minimal depth (the overall mean minimal depth shown in Figure 11).

**Figure 13:**
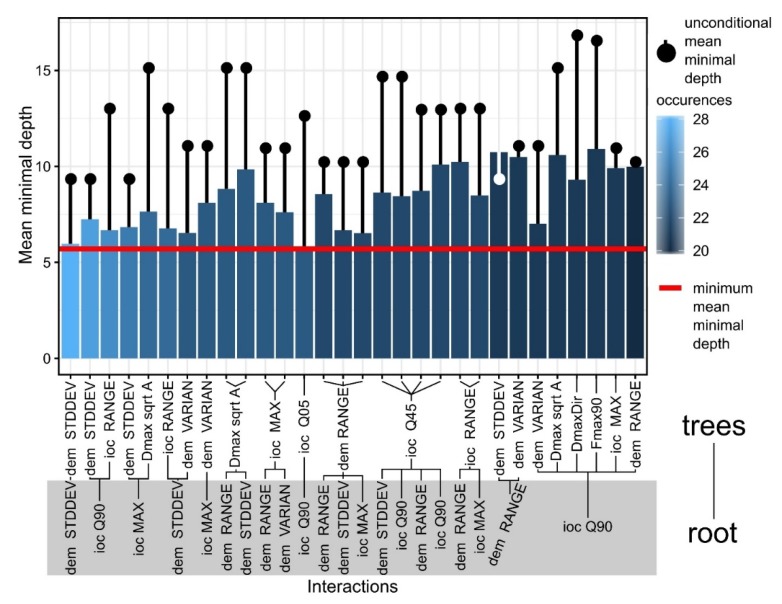
Mean minimal depth for the 30 most frequent interactions. (a high-resolution version can be found at https://doi.org/10.6084/m9.figshare.11798679).

In Figure 13, I show the 30 most frequent interactions of the first five most important variables sorted by the decreasing number of occurrences (color of the bar), the bar showing the mean conditional minimal depth and the line the unconditional mean minimal depth. The standard deviation of altitude had the biggest occurrence, and the unconditional mean minimal depth of it in the forest was almost equal with its mean minimal depth across maximal subtrees. Beside altitude standard deviation, range and variance, which are measures of local peak amplitude, index of convergence measures (maximum and percentiles) define convex features that have local prominence. Shape descriptors appeared to be used for node splitting and interacting with the previous variables at a certain point, the following being used: the ratio between the diameter and square root of area (a measure of similarity to a square, since square root of area, is the side length), Feret diameter measured at an angle of 90° to that of the maximum Feret diameter and shape index.

**Figure 14:**
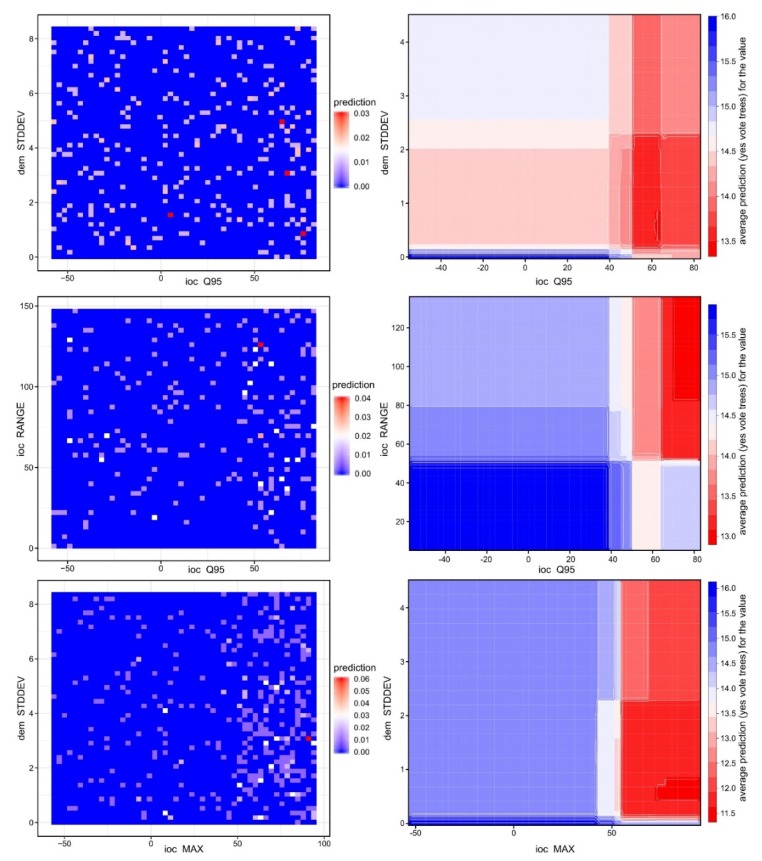
Prediction plots (**left**) and partial dependence plots (**right**) for the most three important variables. (a high-resolution version can be found at https://doi.org/10.6084/m9.figshare.11798682.v1).

**Figure 15:**
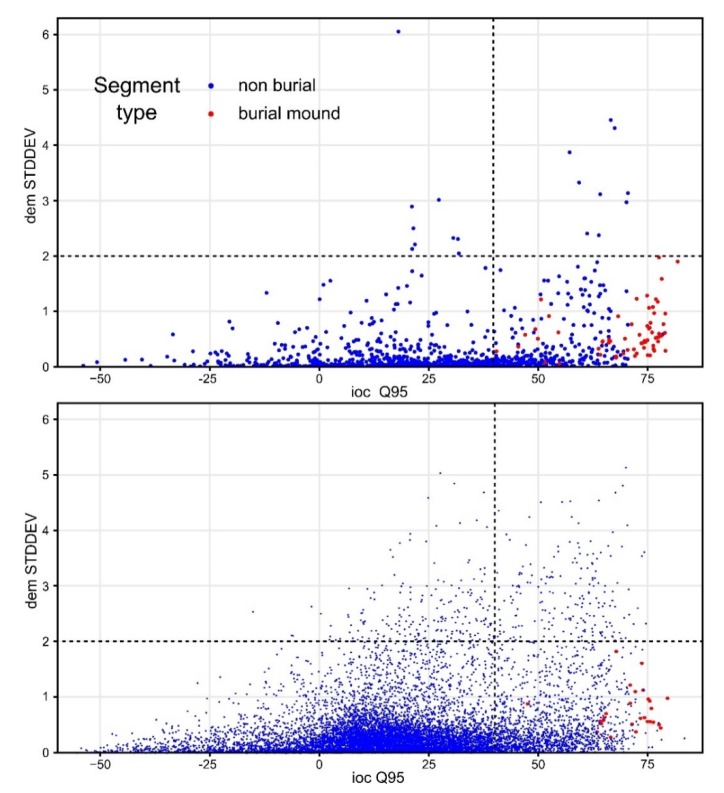
The training (**top**) and the validation (**bottom**) datasets distribution in the feature space of the two most important variables. (a high-resolution version can be found at https://doi.org/10.6084/m9.figshare.11798688.v1).

**Figure 16:**
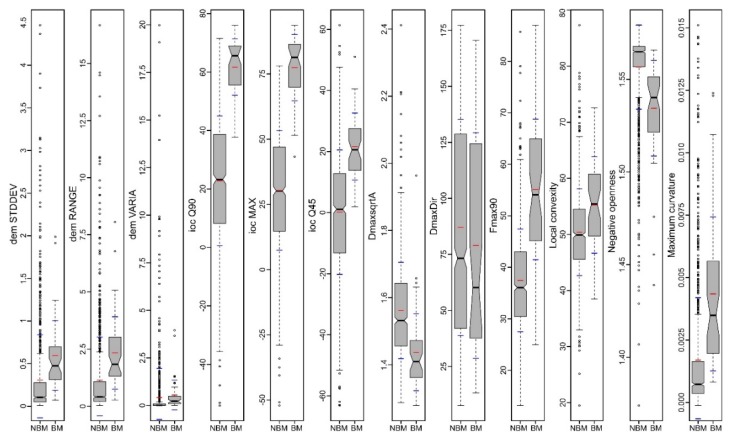
The descriptive statistics of the non-burial mound (NBM) and burial mound (BM) segments for the most important variables and for some variables that were not selected for fitting the RF model (local convexity, negative openness and maximum curvature). (a high-resolution version can be found at https://doi.org/10.6084/m9.figshare.11798691.v1).

In Figure 14 and Figure 15, it can be seen that the feature space of burial mound segments was very well defined, especially for the most important variables. By the examination of the descriptive statistics (Figure 16) for the two segment classes of the eight important variables and three variables that were rejected (non-burial and burial), it can be observed that all of them show statistically significant differences between the classes. This situation shows that the selection of the variables can be made based on expert opinion, but the only by iterative tuning the variables that give the best results can be chosen.

**Figure 17:**
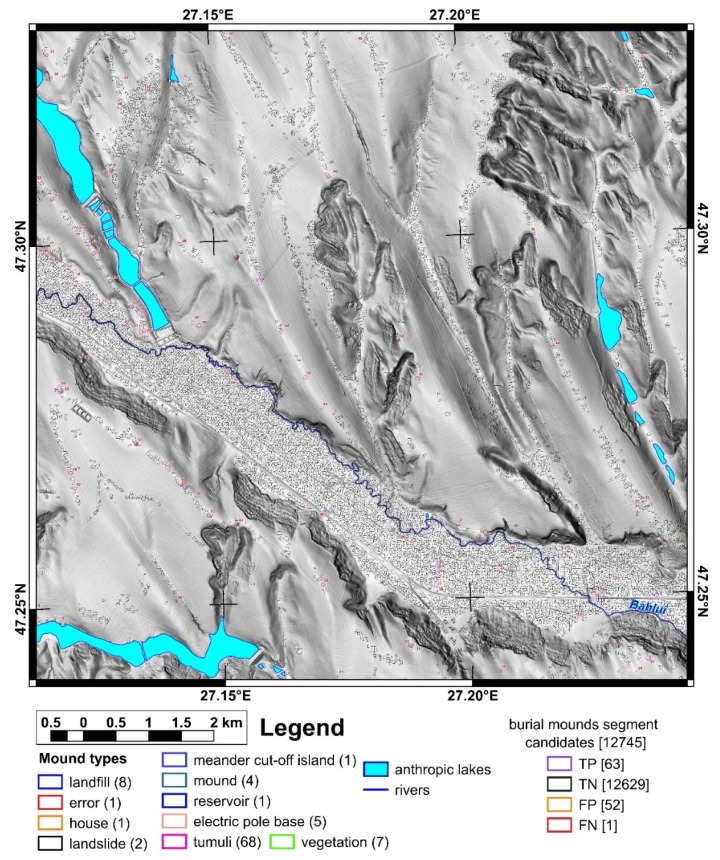
The burial mounds local convexity watershed segments candidates for the northern study area. (a high-resolution version can be found at https://doi.org/10.6084/m9.figshare.11798694.v2).

**Figure 18:**
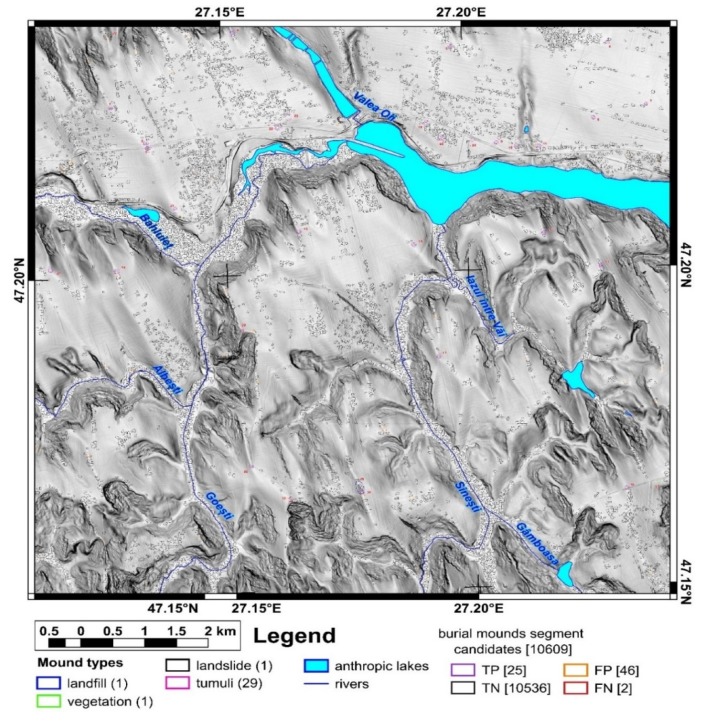
The burial mounds local convexity watershed segments candidates for the validation (southern) study area. (a high-resolution version can be found at https://doi.org/10.6084/m9.figshare.11798697.v1).

In the literature, several approaches are used for training and testing: (i) retaining random 50% for training and validating on the other 50%, (ii) using random 75% for training and the rest 25% for testing, (iii) using cross-validation or (iv) using stratified sampling. The Latin hypercube sampling method was chosen for sampling for the training dataset to supplement the class imbalance. It can be seen in Figure 15 how the training dataset is distributed compared with the validation area. I tested three burial-mounds class frequencies (50%, 75% and 100%) within the training sample. The validation was performed internally on the whole northern dataset (Figure 17 and Table 1) and externally on the whole southern dataset (Figure 18 and Table 1).

As the frequency of burial mound class from the training sample decreased, the precision increased due to fewer false positives, but because the false negative cases also increased, the sensitivity (SNS) and accuracy (ACC) degraded, nonetheless 36% of the burial mounds for southern study area were missed. If the frequency of the burial mound class from the training sample increased, the false positive cases would increase, but also the false negative cases would decrease, situations although when the accuracy is lower, only 9% of burial mounds would be missed. In this situation, there would be 56 segments to be checked in order to find the 82% predicted burial mounds. 

The situation needs to be resolved through a trade-off between how many false positive are enough in order to have the biggest number of true positives. In this regard, depending on the physiographic and anthropic characteristics of the studied area, many non-burial mounds candidates can be filtered if there is the knowledge that burial mounds were not built there (like under the forest, built-up, rocky or swampy areas). The rest of the false positives need to be checked in order to reveal the true positives. Even in this situation, if these segments were clustered (as in the case of the present study, see Figure 17 and Figure 18), the task might not be very time consuming and could be semiautomatized.

### 4.4. Burial Mounds Delineation

Watershed segmentation is able to identify local maxima as seed points and grow the region around until a certain threshold is met. This approach delineates well the convex feature of the mounds and is used in conjunction with the selected peaks to select segments as candidates for burial mounds. Since the majority of the burial mounds have local convexity segments centered around the peak, for validation purposes the centroid of the burial mound was used to identify the association between segments and burial mounds. Only burial mounds that have geomorphometric similarity with shoulders fail to have a centered segment (for example, burial mound 23 from the southern study area).

**Figure 19:**
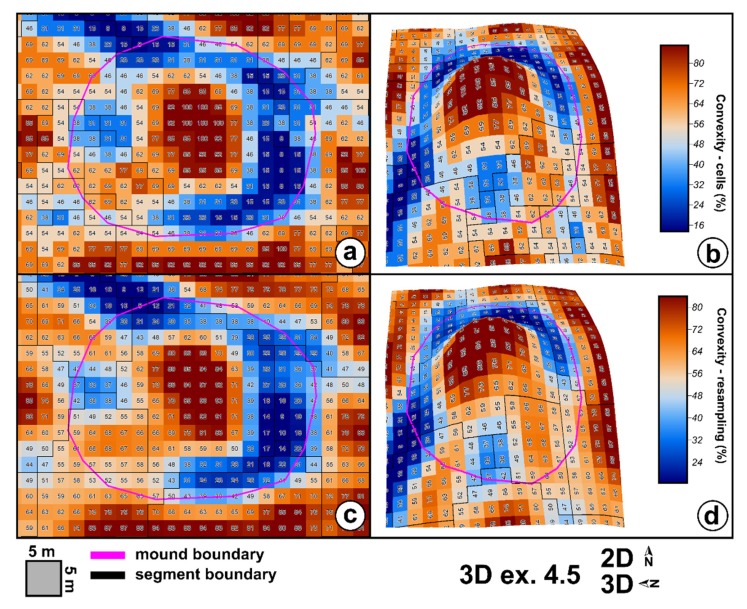
The burial mound watershed delineation based on both methods of local convexity computation: (**a**) (2D) and (**b**) (3D) for cell count method, and (**c**) (2D) and (**d**) (3D) for the cell count interpolation method. (a high-resolution version can be found at https://doi.org/10.6084/m9.figshare.11798700.v1).

For the spatial extension of the burial mounds, the local convexity watershed segmentation works better in its interpolated approach (Figure 6a,b). It can be seen in Figure 6a,b,e,f that the cell count method watershed segment continued beyond the basal edge of the burial mounds along the local ridge. The watershed segments computed with the resampling cell count method delineated pretty well the burial mounds. In Figure 19, it can be seen that this was due to the values of local convexity maxima and minima. While the cell count method gave 100% local convexity around the mound peak, the cell count resampling only gave maxima of 97%. The minima values were repetitive along the mound border for the cell count method (Figure 6a, the western boundary of the mound) and the watershed algorithm did not close the segment, while for the cell count resampling had a variation that was enough for the watershed algorithm to close the segment.

In order to estimate the accuracy of the watershed segmentation regarding the mound delineation, I used the area of the intersection between the watershed segment with the burial mounds manual delineation. 

**Figure 20:**
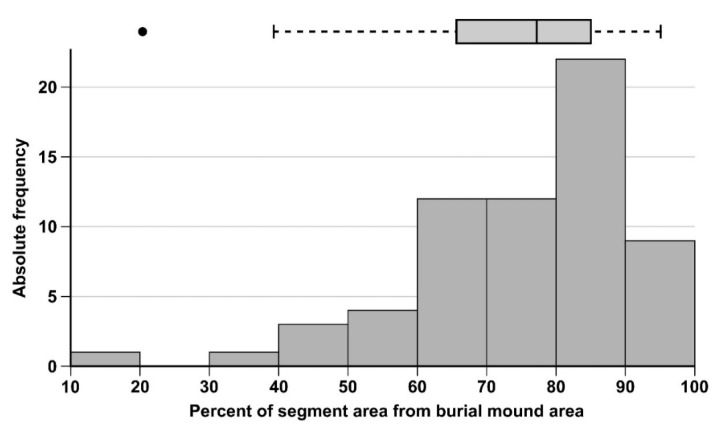
The accuracy statistics of the watershed segmentation for the northern study area. (a high-resolution version can be found at https://doi.org/10.6084/m9.figshare.11798703.v1).

This area was reported as the percentage from the manually delineated burial mounds area. The results for the northern dataset are shown in Figure 20. The distribution if skewed to the right, with only three values under 50%, a mean of 75% and a median of 79% (burial mounds 39 and 38 from the northern study area—Figure 17, having the smallest segment proportions). The watershed segmentation identified very well the convex area of the burial mounds, but not so well the basal part, which was concave.

## 5. Discussion

The proposed method for burial mound identification from high-resolution LiDAR DEM worked in three steps. In the first step, local peaks were identified, and the algorithm missed two burial mounds that due to smoothing did not have peaks (burial mound 85 and 90 from Figure 1, Figures S9 and S10). These two burial mounds were located on inclined hillslopes and, due to smoothing, did not preserve the peak, which was rather a shoulder (as the topographic cross-section in S9 and S10 clearly show). The situation repeats for a landslide body mound, which was mapped (mound 14 from Figure 1). In the southern study area from 29 burial mounds, one burial mound (burial mound 24 from Figure 2 and Figure S12) had the same smoothed appearance, being located on gently inclined hillslopes and having a shoulder shape. Burial mound 24 had its morphology also affected by a road cut.

The peak selection missed another two burial mounds. (burial mounds 55 and 76 from Figure 1, Figures S6 and S8). Burial mound 55 was located on a low steepness (glacis) surface between the Bahlui valley hillslope and its floodplain, had approximately 40 m in diameter and was smoothed (0.75 m in height). Burial mound 76 was located on a furrow ridge on the Bahlui floodplain, had approximately 30 m in diameter and was smoothed (0.75 m in height). Both burial mounds had peaks but not seeds, so no selected peaks. It could be concluded that these burial mounds were missed due to a low height and because they were smoothed by tillage. Another landslide mound, the one with ID 1 from Figure 1 and Figure S1, had a peak, but not a local convexity seed in proximity. In the southern study area, burial mound 23 did not have a selected peak that corresponds to local convexity segment. Its height was 0.6 m, and selected peaks were located on the northern part, but corresponded to segments too small to be included in the burial mound centroid. The shape of this mound was very similar to a shoulder.

The two steps had an accuracy of 96% in identifying the burial mounds. The RF classification was able to find all the remaining 64 burial mounds trained with all the burial mound segments and 63 burial mounds trained with 75% of the burial mound segments; 47 and 52 false positive burial mounds were also found (Figure 17). The validation of the method in the southern study area, of the same size and where 25 burial mounds were selected shows a sensitivity of 0.93 (Table 1), with the tradeoff that 42 and 46 false positive burial mounds were also found.

For the northern study area, burial mound 52 failed to be identified as a burial mound. This burial mound had geomorphometric characteristics above the mean but was not the highest or the biggest. Its detection failure in the scenario of 75% burial mound segments used for training of RF, could be associated with its very concave border, which generated a higher dispersion of the index of convergence statistics.

For the southern study area, burial mounds 29 and 32 (Figure 2 and Figure 18 and Figure S14) failed to be identified. Burial mound 29 was located at the edge of the ridge, a part of it being affected by a landslide scarp, a situation that was already identified in the study area [63] as characteristic for certain burial mound locations. This position makes the corresponding segment geomorphometric characteristics different than the burial mound segments used for training, which are located on flat or gentle slope surface. Burial mound 32 had its morphology degraded by a road cut and by anthropic excavations, deviating from the geomorphometric signature of burial mounds.

The present approach is different from the ones existing in the literature until now [37,39] because it uses both supervised and unsupervised methods, and both pixels and objects. I believe that the use of template matching [17] methods or convolutional neural networks [15] in our case was restricted by (i) the big area of study (100 km^2^) with an uneven repartition of burial mounds and the bigger area, which should be investigated in the future (the whole Moldavian Plateau, approximately 19,000 km^2^). The use of other supervised classification algorithms [39] has the same limitations given by the size of the area. The use of pixel-based variables and geomorphometric objects reduce the computational load and simplify the detection approach.

The usage of a DEM at a 5 m resolution is argued for obtaining shorter run times on normal PCs and for applying the methodology on extended areas. At 5 m spatial resolution, the shape of burial mounds is well represented, and 5 × 5 pixels window fit spatially the smallest burial mound (Figure 7). It is to be expected that the methodology could be used at lower or higher spatial resolutions if the burial mounds have different diameters, but at different spatial resolution the present RF model is unusable, a new RF needs to be fitted again since the scale of the segments will change and the morphometric signature of the burial mounds is different.

Since the problem, from a statistical point of view, is a binary one, actually, the overall accuracy is not necessarily a good measure in practice, but the measures of different types of errors need to be studied. Besides true positives, false positives are also of interest, sensitivity and false positive rate measures of the confusion matrix becoming the most informative regarding the predictive power of the proposed method. Since the shape of the burial mounds is not always the initial one (the height and the diameter have a large range of variation), due to erosion, many false positive cases are found. These false positive cases appear due to similarities in geomorphometry of the burial mounds and of rugged landform facets. The selected segments shown in Figure 17 and Figure 18 are located on floodplains, hillslopes and ridges, in areas where there is a certain convexity, very often in relation with human-induced topography: road embankments and furrows. In this situation, the RF algorithm is able to classify the burial mounds with good accuracy, but at the cost of finding also false positives. Lowering the false positive cases is possible, but comes with a loss of almost 50% of the true positives. The removal of very small and very big segments, dams and embankments lowers the number of false positives (by a half), but does not affect the true positives and negatives.

Compared to the approach of [36], which appears to be the single one in the literature where the study area is large but where there is no specification in which area the accuracy was computed, our approach performs better in terms of accuracy by identifying almost all the mapped burial mounds (Table 1). Compared to the list compiled by [15], our results rank the proposed method as the second, after the one in [41] in terms of confusion matrix accuracy. Unfortunately, [41] does not specify clearly what was the reference dataset and how it was chosen, and presents a confusion matrix that adds up to 25,165 pixels, clearly only a subset of the study area. In this context it is hard to compare it with my method. Even the usage of pixels instead of objects is problematic, since the use of confusion matrix indices is biased by the big number of false positive pixels. Beside the accuracy, which is an overall measure, the numbers of false positive cases that need to be checked by manual intervention for the selection of the burial mounds are important. In my case, the predicted polygons can be automatically mapped on LiDAR DEMs shading and presented to an operator for validation.

Further steps that can improve the method and its applicability will be the testing for the entire Moldavian Plateau, for other areas, where different types of burial mounds and with different spatial scales are present. Since the proposed algorithm can be implemented in open source software, it can be run in a tiled scheme for big areas on high to medium resolution data (1–5 m) and on PCs (4–8 cores and 10–24 GB of memory). The testing of other geomorphometric variables and other automatic segmentation approaches could derive better mapping of burial mound candidates. The proposed approach statistical classification method could also be changed.

## 6. Conclusions

The proposed method based on geomorphometric processing of LiDAR DEMs is able to identify the peaks of burial mounds and to extract with high accuracy their upper part, which is the best-preserved area of the initial burial mound surface. The accuracy of the proposed method is one of the best reported in the literature. The use of geomorphometrical objects (peaks and segments) reduces the feature space of the classifications approach, which is highly imbalanced. Burial mounds are very similar in geomorphometry with other anthropic and natural mounds, but have lower density. The use of the RF approach for the final classification step proved to give satisfactory results since RF is able to resolve the imbalanced class problem and the geomorphometric convergence. RF is able to depict slight geomorphometric signatures that differentiate the burial mounds from other mounds.

The sensitivity of 0.93 and false positive rates of 0.004 are achievable with RF tuning and variable selection, which is recommended for every statistical classification approach. The OBB error is not necessarily a good measure of model performance on the validation dataset, the confusion matrix measures giving the final answer. A good balance between false and true positives needs to be achieved. Nevertheless, a big number of false positives show that the results need to be checked by an operator. The searching area is limited to 1% of the study areas, which is a big improvement compared to a raw search approach. The presented method has the potential to be used on wide areas in order to identify the presence of burial mounds, as is the case of the Moldavian Plateau, and should be tested against other methods and tested on other geomorphometric and archaeological settings.

## Figures and Tables

**Table 1:** The Random Forest (RF) model parameters and the confusion matrix of the validation results.

No	Size of Training Dataset	% Burial Mounds	OBB Error	Northern Study Area				Southern Study Area						
				Confusion Matrix				Confusion Matrix and Measures						
				TP	TN	FP	FN	TP	TN	FP	FN	SNS	FPR	ACC
1	2000	50	1.5	54	12,670	11	10	16	10,572	10	11	0.59	0.001	1
2	2000	75	2	61	12,669	12	3	20	10,571	11	7	0.74	0.001	1
3	2000	100	1.8	64	12,668	13	0	21	10,565	17	1	0.95	0.002	1
4	1000	50	3.3	51	12,663	18	13	17	10,558	24	10	0.63	0.002	1
**5**	**1000**	**75**	**3.1**	**63**	**12,629**	**52**	**1**	**25**	**10,536**	**46**	**2**	**0.93**	**0.004**	**1**
**6**	**1000**	**100**	**3.1**	**64**	**12,634**	**47**	**0**	**25**	**10,540**	**42**	**2**	**0.93**	**0.004**	**1**
7	500	50	5.2	53	12,562	119	11	19	10,487	95	8	0.7	0.009	0.99
8	500	75	4.6	63	12,502	179	1	25	10,382	200	2	0.93	0.019	0.98
9	500	100	3.8	64	12,424	257	0	26	10,279	303	1	0.96	0.029	0.97
10	100	50	10	64	11,337	1344	0	27	9635	947	0	1	0.089	0.91
11	100	75	9	64	11,366	1315	0	27	9662	920	0	1	0.087	0.91
12	100	100	6	64	10,119	2562	0	27	8953	1629	0	1	0.154	0.85

## Supplementary Materials

The following are available online, Figures S1–S9 Burial mounds and other types of mound for the northern study area (https://doi.org/10.6084/m9.figshare.11798715.v1, https://doi.org/10.6084/m9.figshare.11798733.v1, https://doi.org/10.6084/m9.figshare.11798757.v1, https://doi.org/10.6084/m9.figshare.11798766.v1, https://doi.org/10.6084/m9.figshare.11798775.v1, https://doi.org/10.6084/m9.figshare.11798802.v1, https://doi.org/10.6084/m9.figshare.11798811.v1, https://doi.org/10.6084/m9.figshare.11798814.v1, https://doi.org/10.6084/m9.figshare.11798817.v1), Figures S10–S14: Burial mounds and other types of mound for the southern study area (https://doi.org/10.6084/m9.figshare.11798820.v1, https://doi.org/10.6084/m9.figshare.11798823.v1, https://doi.org/10.6084/m9.figshare.11798826.v1, https://doi.org/10.6084/m9.figshare.11798832.v1, https://doi.org/10.6084/m9.figshare.11798835.v1), Table S1: The list of geomorphometrical variables and their computation settings in SAGA GIS (https://www.mdpi.com/1424-8220/20/4/1192/s1), Table S2: The list of the geomorphometrical variables and shape descriptors used for fitting the RF model (https://www.mdpi.com/1424-8220/20/4/1192/s1).
