# Supplementary material for: Geomorphometric Methods for Burial Mound Recognition and Extraction from High-Resolution LiDAR DEMs
Source: Sensors (Basel). 2020 Feb 21;20(4):1192. doi: 10.3390/s20041192 (PMC7070870; doi:10.3390/s20041192)
Supplement: Supplementary file 1 [file sensors-20-01192-s001.zip › Table S1.docx]

**Table S1.** The list of geomorphometrical variables and their computation settings in SAGA GIS.

| **No** | **Code** | **Name** | **Description** | **SAG GIS module and function** | **Parameters of computation*** |
| --- | --- | --- | --- | --- | --- |
|  |  |  |  |  |  |
| 1 | A | Area | Polygon area | Shapes / Polygon / Polygon Shape Indices  - | |
| 2 | P | Perimeter | Polygon perimeter |  |  |
| 3 | P.A | Interior edge ratio | P/A |  |  |
| 4 | P.sqrt.A. |  | P/sqrt(A) |  |  |
| 5 | Depqc | Equivalent projected circle diameter | 2*sqrt(A/π) |  |  |
| 6 | Sphericity | Sphericity | The ratio of the perimeter of the equivalent circle to the real perimter |  |  |
| 7 | Shape.Index | Shape index | Inverse of Sphericity |  |  |
| 8 | Dmax | Maximum diameter | Maximum distance between two polygon part’s vertices |  |  |
| 9 | DmaxDir | Direction of maximum diameter | |  |  |
| 10 | Dmax.A | Dmax/A | |  |  |
| 11 | Dmax.sqrt.A | Dmax/sqrt(A) | |  |  |
| 12 | Dgyros | Diameter of gyration | Twice the maximum vertex distance to its polygon part’s centroid |  |  |
| 13 | Fmax | Maximum Feret diameter | |  |  |
| 14 | FmaxDir | Direction of the maximum Ferret diameter | |  |  |
| 15 | Fmin | Minimum Feret diameter | |  |  |
| 16 | FminDir | Direction of the minimum Feret diameter | |  |  |
| 17 | Fmean | Mean Feret diameter | |  |  |
| 18 | Fmax90 | Feret diameter measured at an angle of 90° to that of the Fmax direction | |  |  |
| 19 | Fmin90 | Feret diameter measured at an angle of 90° to that of the Fmin direction | |  |  |
| 20 | Fvol | Diameter of a sphere having the same volume as the cylinder constructed by Fmin as the cylinder diameter and Fmax as its length | |  |  |
| 21 | dem | Elevation |  |  |  |
| 22 | ioc | Index of convergence |  | Terrain Analysis / Morphometry / Convergence Index (Search Radius) | Radius=5 |
| 23 | conv_r | Local convexity |  | Terrain Analysis / Morphometry / Convergence Index (Search Radius) | Gradient=0, Difference=direction to the center cell, Radius=2 |
| 24 | nego | Negative openness |  | Terrain Analysis / Morphometry / | Radial Limit=100, Method=line tracing, Number of Sectors=8, Unit=Degrees, Difference from Nadir=1 |
| 25 | slop | Slope |  | Terrain Analysis / Morphometry / Morphometric Features | Scale radius (Cells)=2, Constrain=1 |
| 26 | proc | Profile curvature |  |  |  |
| 27 | plac | Plan curvature |  |  |  |
| 28 | logc | Longitudinal curvature |  |  |  |
| 29 | croc | Cross-sectional curvature |  |  |  |
| 30 | minc | Minimum curvature |  |  |  |
| 31 | maxc | Maximum curvature |  |  |  |
| 32 | rare | Real surface area |  | Terrain Analysis / Morphometry / Real Surface Area | default |
| 33 | wind | Wind exposition index |  | Terrain Analysis / Morphometry / Wind Exposition Index | Search Distance [km]=0.1, Angular Step Size (Degree)=15, Elevation Averaging=1 |
| 34 | tpi | Topographic position index |  | Terrain Analysis / Morphometry / Multi-Scale Topographic Position Index (TPI) | Minimum Scale=1, Maximum Scale=8, Number of scales=8 |
| 35 | vld | Valley depth |  | Terrain Analysis / Morphometry / Relative heights and Slope Positions | default |
| 36 | mpi | Morphometric protection index |  | Terrain Analysis / Morphometry / Morphometric Protection Index | Radius=100 |
| 37 | tri | Terrain ruggedness index |  | Terrain Analysis / Morphometry / Terrain Ruggedness Index (TRI) | Search Mode=Circle, Search Radius=2 |
| 38 | vrm | Vector ruggedness measure |  | Terrain Analysis / Morphometry / Vector Ruggedness Measure (VRM) | Search Mode=Circle, Search Radius=2 |
| 39 | txt | Terrain surface texture |  | Terrain Analysis / Morphometry / Terrain Surface Texture | Flat Area Threshold=0, Scale(cells)=2, Method=resampling |
| 40 | clo | Local curvature |  | Terrain Analysis / Morphometry / Upslope and Downslope Curvature | Upslope weighting=0.5 |
| 41 | cup | Upslope curvature |  |  |  |
| 42 | clu | Local upslope curvature |  |  |  |
| 43 | cdo | Downslope curvature |  |  |  |
| 44 | cdl | Local downslope curvature |  |  |  |
| 45 | flo | Flow accumulation |  | Terrain Analysis / Hydrology / Flow Accumulation (Recursive) | Flow Accumulation Unit=number of cells, Method=Multiple Flow Direction |
| 46 | fpl | Flow path length |  | Terrain Analysis / Hydrology / Flow Path Length | Method= Multiple Flow Direction (FD8) |
| 47 | spl | Slope length |  | Terrain Analysis / Hydrology / Slope Length | default |
| 48 | cbl | Cell balance | Ratio between flow input and output | Terrain Analysis / Hydrology / Cell Balance | Method=Multiple Flow Direction |
| 49 | twi | Topographic wetness index | SAGA implementation of TWI using a modified catchment area, that is more realistic, compared to standard TWi | Terrain Analysis / Hydrology / SAGA Wetness Index | default |
| 51 | dhratio | Diameter-height ratio | Dmax/dem RANGE | - | - |
| 52 | compactness | Compactness | (Sqrt(4*(A/π)))/P | - | - |
| 53 | formfactor | Form factor | (4*π*A)/(P/2) | - | - |
| 54 | roundness | Roundness | (4*A)/(π*Fmax) | - | - |
| 55 | elongation | Elongation | Fmax/Fmin | - | - |

*The full description of the functions and parameters can be found here <http://www.saga-gis.org/saga_tool_doc/7.0.0/a2z.html>
