# Supplementary material for: Geomorphometric Methods for Burial Mound Recognition and Extraction from High-Resolution LiDAR DEMs
Source: Sensors (Basel). 2020 Feb 21;20(4):1192. doi: 10.3390/s20041192 (PMC7070870; doi:10.3390/s20041192)
Supplement: Supplementary file 1 [file sensors-20-01192-s001.zip › Table S2.docx]

**Table S2.** The list of geomorphometrical variables and their computation settings in SAGA GIS.

| **No** | **Code** | **Name** |  |
| --- | --- | --- | --- |
|  |  |  |  |
| 1 | A | Area |  |
| 2 | P | Perimeter |  |
| 3 | P.A | Interior edge ratio |  |
| 4 | P.sqrt.A. |  |  |
| 5 | Depqc | Equivalent projected circle diameter |  |
| 6 | Sphericity | Sphericity |  |
| 7 | Shape.Index | Shape index |  |
| 8 | Dmax | Maximum diameter |  |
| 9 | DmaxDir | Direction of maximum diameter  Dmax/A | |
| 10 | Dmax.A |  |  |
| 11 | Dmax.sqrt.A |  |  |
| 12 | Dgyros | Diameter of gyration |  |
| 13 | Fmax | Maximum Feret diameter | |
| 14 | FmaxDir | Direction of the maximum Ferret diameter | |
| 15 | Fmin | Minimum Feret diameter | |
| 16 | FminDir | Direction of the minimum Feret diameter | |
| 17 | Fmean | Mean Feret diameter | |
| 18 | Fmax90 | Feret diameter measured at an angle of 90° to that of the Fmax direction | |
| 19 | Fmin90 | Feret diameter measured at an angle of 90° to that of the Fmin direction | |
| 20 | Fvol | Diameter of a sphere having the same volume as the cylinder constructed by Fmin as the cylinder diameter and Fmax as its length | |
| 21 | dem MIN | Minimum elevation |  |
| 22 | dem MAX | Maximum elevation |  |
| 23 | dem RANGE | Range of elevation |  |
| 24 | dem SUM | Sum of elevation |  |
| 25 | dem MEAN | Mean elevation |  |
| 26 | dem VARIAN | Elevation variance |  |
| 27 | dem STDDEV | Standard deviation of elevation |  |
| 28 | dem Q05 | Elevation percentiles, multiples of 5 |  |
| 29 | dem Q10 |  |  |
| 30 | dem Q15 |  |  |
| 31 | dem Q20 |  |  |
| 32 | dem Q25 |  |  |
| 33 | dem Q30 |  |  |
| 34 | dem Q35 |  |  |
| 35 | dem Q40 |  |  |
| 36 | dem Q45 |  |  |
| 37 | dem Q50 |  |  |
| 38 | dem Q55 |  |  |
| 39 | dem Q60 |  |  |
| 40 | dem Q65 |  |  |
| 41 | dem Q70 |  |  |
| 42 | dem Q75 |  |  |
| 43 | dem Q80 |  |  |
| 44 | dem Q85 |  |  |
| 45 | dem Q90 |  |  |
| 46 | dem Q95 |  |  |
| 47 | ioc MIN | Minimum index of convergence |  |
| 48 | ioc MAX | Maximum index of convergence |  |
| 49 | ioc RANGE | Range of index of convergence |  |
| 51 | ioc SUM | Sum of index of convergence |  |
| 52 | ioc MEAN | Mean index of convergence |  |
| 53 | ioc VARIAN | Index of convergence variance |  |
| 54 | ioc STDDEV | Standard deviation of index of convergence |  |
| 55 | ioc Q05 | Indexs of confergence percentiles, multiples of 5 |  |
| 56 | ioc Q10 |  |  |
| 57 | ioc Q15 |  |  |
| 58 | ioc Q20 |  |  |
| 59 | ioc Q25 |  |  |
| 60 | ioc Q30 |  |  |
| 61 | ioc Q35 |  |  |
| 62 | ioc Q40 |  |  |
| 63 | ioc Q45 |  |  |
| 64 | ioc Q50 |  |  |
| 65 | ioc Q55 |  |  |
| 66 | ioc Q60 |  |  |
| 67 | ioc Q65 |  |  |
| 68 | ioc Q70 |  |  |
| 69 | ioc Q75 |  |  |
| 70 | ioc Q80 |  |  |
| 71 | ioc Q85 |  |  |
| 72 | ioc Q90 |  |  |
| 73 | ioc Q95 |  |  |
